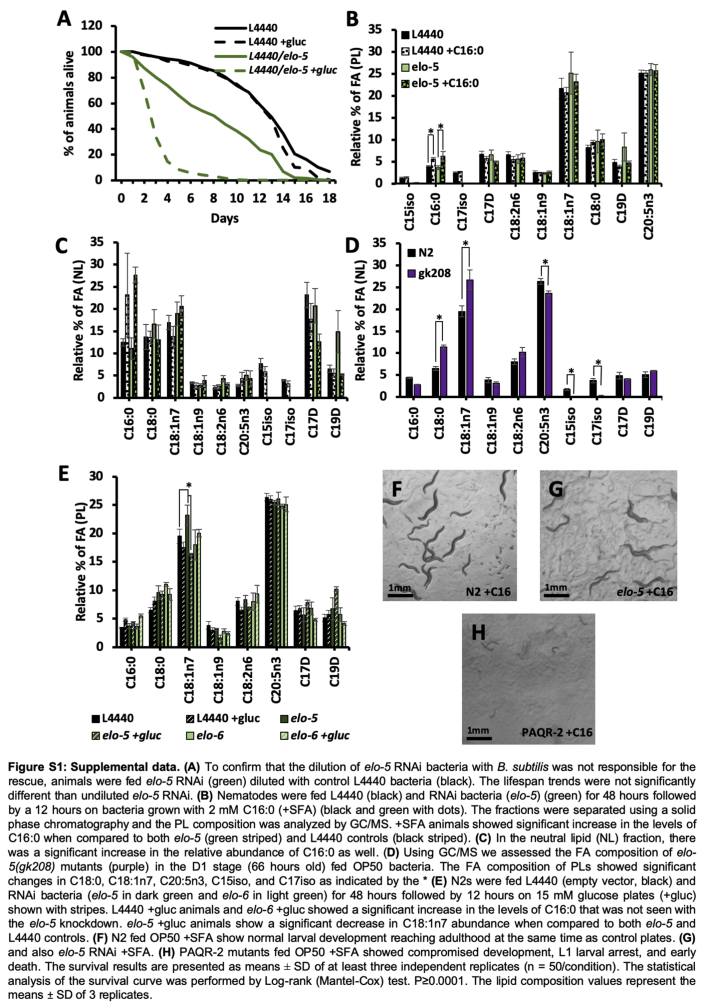# Monomethyl branched-chain fatty acids are critical for *Caenorhabitis elegans* survival in elevated glucose conditions

**DOI:** 10.1016/j.jbc.2021.101444

**Published:** 2021-11-23

**Authors:** Andre F.C. Vieira, Mark A. Xatse, Hamide Tifeki, Cédric Diot, Albertha J.M. Walhout, Carissa Perez Olsen

**Affiliations:** 1 Department of Chemistry and Biochemistry, Worcester Polytechnic Institute, Worcester, Massachusetts, USA; 2 Department of Chemistry, University of Alaska Anchorage, Anchorage, Alaska, USA; 3 Program in Systems Biology and Program in Molecular Medicine, UMASS Medical School, Worcester, Massachusetts, USA

**Keywords:** membranes, phospholipid, mass spectrometry, stable isotopes, glucose response, GC-MS, gas chromatography–mass spectrometry, mmBCFA, monomethyl branched-chain fatty acid, MUFA, monounsaturated fatty acid, PUFA, polyunsaturated fatty acid

## Abstract

The maintenance of optimal membrane composition under basal and stress conditions is critical for the survival of an organism. High-glucose stress has been shown to perturb membrane properties by decreasing membrane fluidity, and the membrane sensor PAQR-2 is required to restore membrane integrity. However, the mechanisms required to respond to elevated dietary glucose are not fully established. In this study, we used a ^13^C stable isotope-enriched diet and mass spectrometry to better understand the impact of glucose on fatty acid dynamics in the membrane of *Caenorhabditis elegans*. We found a novel role for monomethyl branched-chain fatty acids (mmBCFAs) in mediating the ability of the nematodes to survive conditions of elevated dietary glucose. This requirement of mmBCFAs is unique to glucose stress and was not observed when the nematode was fed elevated dietary saturated fatty acid. In addition, when worms deficient in *elo-5*, the major biosynthesis enzyme of mmBCFAs, were fed *Bacillus subtilis* (a bacteria strain rich in mmBCFAs) in combination with high glucose, their survival rates were rescued to wild-type levels. Finally, the results suggest that mmBCFAs are part of the PAQR-2 signaling response during glucose stress. Taken together, we have identified a novel role for mmBCFAs in stress response in nematodes and have established these fatty acids as critical for adapting to elevated glucose.

In many disease states, including diabetes, cancer, and neurodegenerative diseases, defects in membrane structure and composition have been identified (1, 2, 3, 4, 5, 6). Biological membranes are essential barriers between the intracellular and extracellular environment and are also crucial in the compartmentalization of subcellular organelles. Membranes are not limited to establishing passive barriers, but they also influence numerous and diverse cellular bioactivities such as signaling and regulation (7, 8, 9, 10, 11). Although membranes allow a certain degree of variation in their makeup, it is critical to maintain a membrane composition within an acceptable range for optimal cellular function. Several studies have shown that there are regulatory mechanisms, including membrane sensors, that detect perturbations in the biophysical properties of the membrane and can allow membranes to adapt to variations in environmental cues including temperature and dietary composition (12, 13).

Model systems including the nematode, *Caenorhabitis elegans,* have been established in order to probe the regulation of membrane composition and the response to perturbations. In addition to the genetic tools available, *C. elegans* has the added advantage of being small enough to allow for high isotope enrichment and subsequent detailed measurements of membrane flux. Our lab has developed stable isotope feeding strategies in *C. elegans* to track the incorporation of new fatty acid molecules into the membrane (14, 15). Using this technique, we established that the majority of the membrane lipids are replaced or modified within a 24-h period in young animals. This replacement is significantly greater in the phospholipids showing 1.7-fold faster renewal than in neutral lipids used to store fat in the same animals (15, 16). Thus, in addition to responding to external and internal stimuli, the phospholipid membrane is a highly dynamic structure with tremendous flux of lipids in and out of the membrane even under basal conditions. Not only is the turnover greatly abundant in membrane lipids, but the rates differ depending on the specific type of lipid, thus allowing for the maintenance of overall membrane composition and adjustment of that content when needed.

Monomethyl branched-chain fatty acids (mmBCFAs) are present in plants, bacteria, and animals, and decreased levels of mmBCFAs in humans have been linked to obesity and insulin resistance (17). In *C. elegans,* mmBCFAs are found within the membrane at significant concentrations, accounting for about 5.5% of the relative abundance of FAs. They are synthesized from branched-chain amino acids particularly leucine through the activity of the branched-chain ketoacid dehydrogenase complex (BCKDH), fatty acid synthase (FASN-1), and acetyl-coA carboxylase (POD-2) ultimately resulting in the production of C13iso, which is elongated by *elo-5* and *elo-6* to make C15iso and C17iso; (18, 19). mmBCFAs are critical for post embryonic growth facilitated in part by activating TORC1 signaling pathway to facilitate nutrient sensing and metabolism in nematodes (20, 21, 22). In bacteria, the presence of mmBCFAs impacts the packing of the membrane and influences overall membrane fluidity (23, 24). In fact, two branched-chain fatty acid classes, referred to as iso and anteiso, contain a methyl group at either the penultimate or the antepenultimate carbon, respectively, and are the most prominent fatty acids of cell membranes of some bacteria, especially of the genus *Bacillus*. The presence of these branched fatty acids has a different impact on membrane properties such as packing, since they mimic properties of both straight-chain saturated and cis-monounsaturated fatty acids (MUFAs). The ratio iso/anteiso fatty acids is reduced when the growth temperature is decreased, indicating that anteiso species strongly affect membrane fluidity compared with iso species (25, 26). However, the participation of mmBCFAs in the response to other external stress such as high glucose diet has not yet been described.

Altered conditions such as temperature changes require that the membrane composition is adjusted in order to maintain the membrane’s properties. For instance, lower temperatures reduce overall membrane fluidity in poikilotherms and, thus, require an increase in the amount of unsaturated fatty acids provided to the membrane to maintain membrane function (27, 28). The presence of double bonds in the cis configuration causes kinks or bends in the acyl chain, affecting the overall fatty acid packing and consequently the membrane structure increasing fluidity (29). The amount of saturated fat within a membrane must be balanced with monounsaturated (MUFA) and polyunsaturated (PUFA) fatty acids to tune the biophysical properties of the membrane (30). Other fatty acids including mmBCFA contribute to overall membrane fluidity and permeability as well.

Glucose homeostasis is critical, and organisms have multiple regulatory mechanisms that allow them to maintain optimum membrane composition when challenged with glucose. For example, PAQR-2 is a protein capable of sensing and responding to the accumulation of saturated fat within the endoplasmic reticulum induced by cold temperature. Additionally, PAQR-2 is required for survival in other conditions that perturb the membrane properties including excess dietary glucose (31). In the absence of PAQR-2, nematodes fed glucose accumulate SFAs, have a withered tail, and reduced viability. The most well-characterized role for PAQR-2 is to provide UFA to the membrane through the FAT-7 desaturase, which incorporates the first double bond into the saturated fatty acids. However, it is unclear if PAQR-2 influences the activity of other metabolic pathways that contribute to survival in elevated glucose diets. Although it is clear that membrane composition is impacted by glucose stress, the mechanisms involved in responding to perturbations in membrane properties have not been completely defined. In our studies, we use stable isotope labeling under elevated glucose conditions to monitor the fatty acid populations of the membrane after stress. Our results unveiled a novel role for mmBCFAs in surviving elevated glucose conditions. We have probed these relationships to find that nematodes not producing mmBCFAs show decreased survivorship when stressed with glucose.

## Results

### Quantifying membrane dynamics with elevated dietary glucose

While it has been established that high-glucose diets require a compensatory metabolic shift in lipid pathways, the specific alterations in the membrane lipids of animals exposed to excess glucose have not been identified. To define the response to dietary glucose, we transferred larval (L4) *C. elegans* to 15 mM glucose supplementation plates (referred to as “+gluc plates”), which is the concentration where phenotypes are observed in *paqr-2* mutant animals with minimal lethality (13). After 12 h on +gluc plates, animals were collected, fatty acid methyl esters were created from the major lipid classes (*i.e.*, phospholipids, neutral lipids, glycolipids) and analyzed by gas chromatography–mass spectrometry (GC-MS) to quantify the relative abundance of associated fatty acids (see Experimental procedures) (14). We will use the standard nomenclature (CX:YnZ) to describe the fatty acid populations where X indicates the number of carbons in the fatty acid, and Y shows the number of double bonds at position Z for our analysis.

**Figure 1:**
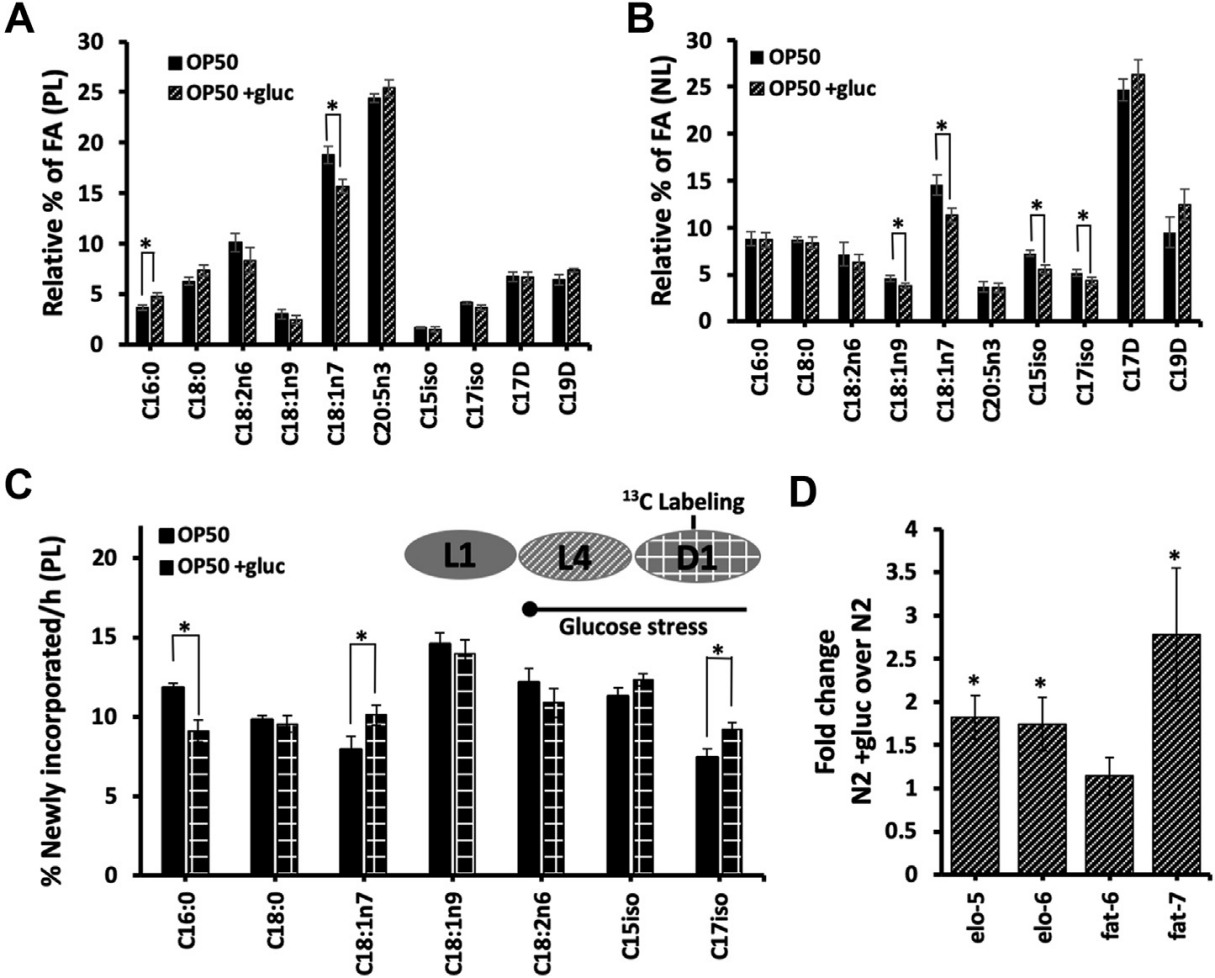
**Wild-type nematodes under glucose stress maintain optimal membrane composition but have altered membrane dynamics.** L1 animals were fed OP50 bacteria on HG plates for 48 h followed by 12 h of OP50 feeding on 15 mM glucose plates (+gluc; indicated throughout with stripes) and an additional 6 h of stable isotope labeling required for part *C*. *A*, FAMEs from the PL population were analyzed by GC/MS and the relative abundance of each fatty acid species was quantified. Here, the major FA species of interest are shown with significant alterations observed only in C16:0 and C18:1n7 of glucose-fed animals (striped) *versus* controls (*black*). *B*, in the isolated neutral lipid (NL) fraction, there were alterations in the relative abundance of four FAs: C18:1n9, C18:1n7, C15iso, and C17iso. *C*, after 15 mM glucose supplementation, animals were fed OP50 bacteria enriched with ^13^C stable isotope in the proportion of 60:40 (^13^C:^12^ C) for 6 h on agarose plates (isotope labeling on +gluc is shown throughout as checkered). The ^13^C-label was used to quantify the newly incorporated fatty acids during the labeling window. Because a 60:40 mixture was used, an adjustment factor was used to define the entire newly incorporated population of lipids and is shown here. There were significantly lower levels of labeled C16:0 and higher levels of labeled C18:1n7 and C17iso in +gluc animals. For all GC/MS analysis, values represent means ± SEM of at least nine replicates. Statistical significance (*p* < 0.05) is indicated by ∗ and was calculated using unpaired T tests and F tests to compare variances. *D*, qRT-PCR was conducted on N2 +gluc and normalized to untreated animals. The expressions of *elo-5, elo-6*, and *fat-7* were significantly upregulated as evaluated by applying a One-Sample *t* test (hypothetical value = 1) on GraphPad Prism (v9.0.0). FAMEs, fatty acid methyl esters.

We first examined the changes in phospholipids on +gluc plates focusing on the major fatty acid populations (>1.5% of the total pool). Here, we do not report the fatty acid analysis for the polyunsaturated fatty acids, because they contain more than two double bonds and fragment extensively in the mass spectrometer making them incompatible with the stable isotope studies reported later. We found that there were no significant changes in any major fatty acid species with the exception of a decrease in vaccenate (C18:1n7) from 18.1 ± 0.8% to 15.7 ± 0.6% and a slight increase in palmitate (C16:0) from 3.7 ± 0.2% to 4.8 ± 0.3% (Fig. 1*A*). The reduction in C18:1n7 levels is likely due to the reduced presence of this species in the bacteria exposed to glucose. The lack of major changes in the phospholipid populations has been previously demonstrated by HPLC-MS-based studies as well (13). The result further supports the presence of maintenance mechanisms to specifically control the fatty acid composition of the membrane lipids under elevated glucose concentrations.

These maintenance pathways may be specific to membrane lipids or may regulate lipid metabolism pathways in general. Therefore, to determine whether glucose supplementation impacts the other pools within the nematode, we evaluated the neutral lipid fraction, which represents the stored fat. After extraction and analysis by GC-MS, we found more significant changes in the overall fatty acid profile from neutral lipids. Specifically, there are significant decreases of multiple fatty acid populations including a reduction of vaccenate (C18:1n7) by 22%, oleate (C18:1n9) by 17%. The mmBCFAs, 13-methyl myristic acid (C15iso), and 15-methyl hexadecanoic acid (C17iso) were decreased by 23% and 17% respectively (Fig. 1*B*). These alterations support the conclusion that glucose supplementation drives changes in lipid metabolism pathways and that the neutral lipid pools are regulated less than phospholipid populations.

One potential issue using the GC/MS to analyze the membrane composition is that changes in lipid pools might be diluted by the preexisting large quantities of lipids at the start of the glucose stress. Therefore, we incorporated a global dietary stable isotope tracer to monitor the different types of lipids provided to the membrane immediately following glucose supplementation. The nematodes were fed bacteria enriched with ^13^C-isotopes by growing the standard laboratory diet (OP50) in Isogro ^13^C-enriched media (>99% ^13^C from Sigma-Aldrich) (15). After the appropriate corrections (defined in the Experimental procedures), the stable isotope labeling was examined in the palmitate (C16:0) population, as previous studies have observed high levels of palmitate in the absence of corrective pathways. There is a significant reduction in the provision of C16:0 to the membrane with 8.6 ± 0.6% of C16:0 being newly incorporated in glucose-fed worms compared with 11.5 ± 0.3% in control animals (Fig. 1*C*). To determine if this decrease is a common feature of saturated fatty acids, we examined C18:0 and found no significant decrease in C18:0 stable isotope patterns. Thus, we conclude that the downregulation of flux is specific to C16:0 and not all saturated fatty acids.

Because of the use of a general stable isotope tracer, we can probe the remaining fatty acid species in the same animals with the exception of the C20 PUFAs that fragment extensively in the mass spectrometer. The dynamics of the C18 mono- and polyunsaturated fatty acids, namely oleate (C18:1n9) and linoleate (C18:2n6), were not impacted by the glucose supplementation (Fig. 1*C*). However, we identified a significant increase in the provision of vaccenate (C18:1n7) and the mmBCFA, C17iso, to the membrane (Fig. 1*C*). This mmBCFA species is not significant component of the diet, and thus this increase likely represents *de novo* synthesis of these fats. Specifically, the labeled population in C18:1n7 increases by 27% and by 23% in C17iso. Overall, most unsaturated membrane lipids are unchanged from control populations, but there is an overall reduction in the labeling of saturated fatty acids and an increase in mmBCFAs following glucose feeding. We hypothesize that these changes may be important responses to the elevated glucose levels.

In order to determine if there is an increase in mmBCFA production, we next examined the expression of the *fat-6* and *fat-7* desaturases and the mmBCFA *elo-5* and *elo-6* elongases after glucose feeding. In previous reports, *fat-7* has been shown to be upregulated with glucose diets, and the qRT-PCR analysis completed here shows a significant increase in expression with glucose feeding (Fig. 1*D*). This upregulation is specific to *fat-7* as *fat-6* mRNA levels are unchanged. There is a significant increase of both *elo-5* and *elo-6* expression by 1.8 and 1.7-fold indicating that these genes are transcriptionally upregulated to promote mmBCFA synthesis in elevated glucose conditions (Fig. 1*D*).

### mmBCFAs are critical for surviving glucose supplementation

**Figure 2:**
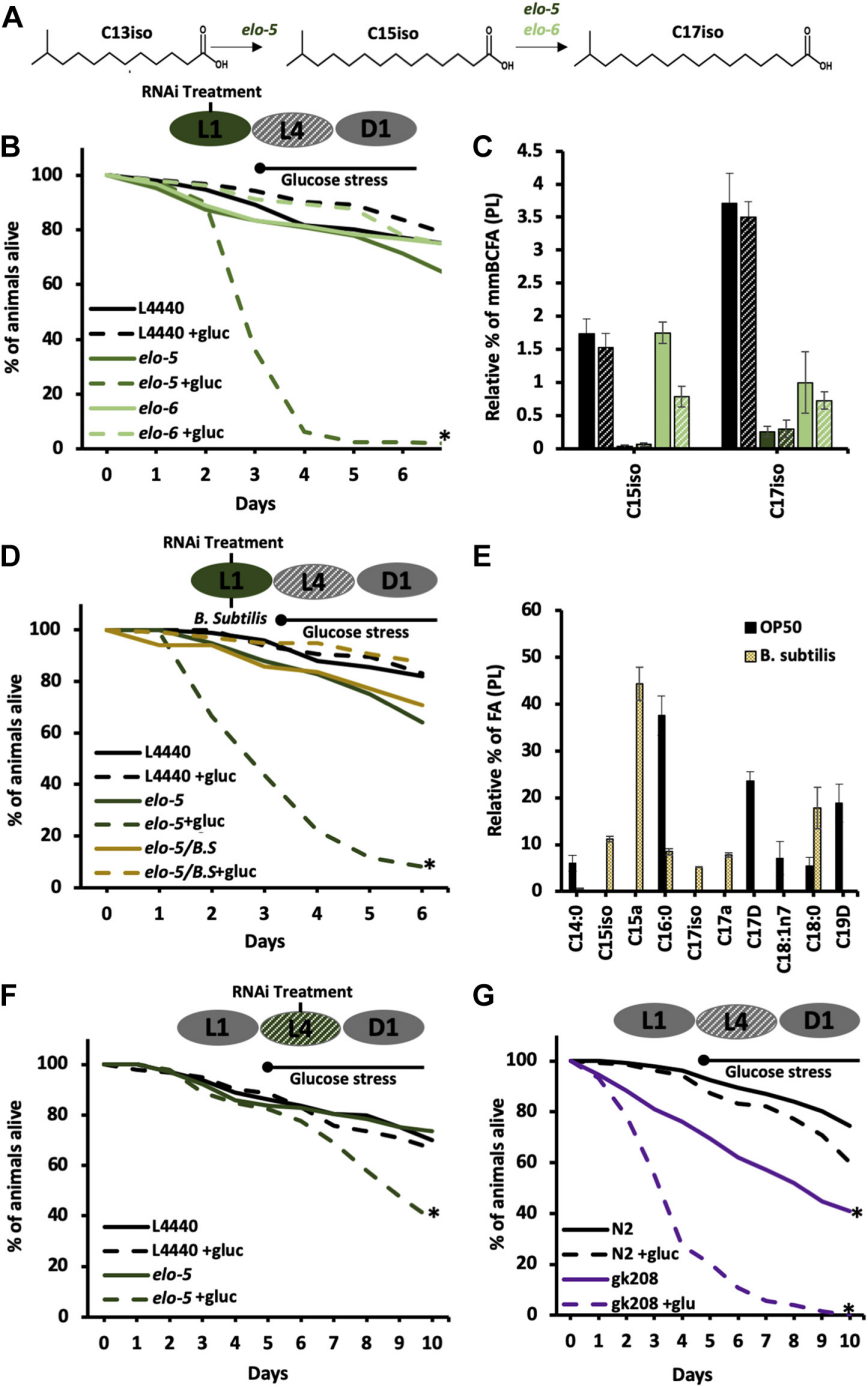
**mmBCFAs are essential for survival with elevated dietary glucose.***A,* the elongases, *elo-5* (*dark green*) and *elo-6* (*light green*), produce the two mmBCFAs (C15iso and C17iso) by elongation steps shown here. *B*, RNAi knockdown of *elo-5* (*dark green*) and *elo-6* (*light green*) in N2 worms started from L1 along with control empty vector RNAi (L4440) (*black*). At the L4 stage, animals were transferred to 45 mM glucose (*dashed lines*), and the percent of animals alive was assessed daily. On day 4, we observed that 94% of the nematodes were dead in *elo-5* RNAi +gluc plates. *C*, The levels of mmBCFAs confirm efficient knockdown with animals fed *elo-5* RNAi on +gluc plates having almost no detected C15iso or C17iso. In *elo-6* animals, there was a significant decrease in the levels of C15iso with on +gluc but no significant difference in C17iso (*light green striped*). *D*, To confirm the role of mmBCFAs in survival on +gluc plates, L1 animals were fed a bacteria strain rich in mmBCFAs (*B. subtilis*) (*yellow*). A ratio of 1:1 (RNAi bacteria to *B. subtilis* ratio) was used to ensure sufficient knockdown of *elo-5*. *E*, FAMEs were analyzed by GC/MS to generate an FA profile of the diet. *B. subtilis* (*yellow*) compared to OP50 (*black*). There are significant differences including elevated C15iso, C15a (anteiso mmBCFA), C17iso, and C17a. *F*, RNAi knockdown of *elo-5* was initiated later and simultaneously with the transfer to +gluc plates. On day 9, 53% death was seen in *elo-5* +gluc animals. *G*, *elo-5(gk208)* mutants (*purple*) were grown on +gluc plates (*dashed line*) and monitored for death daily. *elo-5* mutants have dramatically reduced viability on +gluc plates similar to the *elo-5* RNAi feeding. Survival curves are presented as means of at least three independent replicates with n = 50 nematodes/condition. All the statistical analysis was performed by Log-rank (Mantel-Cox) test. *p* ≥ 0.0001 is represented by ∗. The lipid composition values represent means ± SEM of at least four replicates. Statistical significance (*p* < 0.05) was calculated using an unpaired *t* test and F test to compare variances.

Because the stable isotope labeling shows increased mmBCFA provision to the membrane following glucose supplementation, we tested if mmBCFAs play a role in responding to the elevated glucose conditions. We compromised their production with *elo-5* RNAi because ELO-5 is the elongase essential for the synthesis of both significant mmBCFA species, C15iso and C17iso (Fig. 2*A*). A 12-h feeding period before *elo-5* RNAi treatment was used to prevent the smaller size and shorter life span seen with *elo-5* RNAi initiated immediately at L1. After 48 h of growth, L4 animals were placed on 15 mM +gluc plates as in the previous lipid studies or a higher 45 mM +gluc plate to ensure sufficient glucose exposure. At both glucose concentrations, there was significantly more death in the *elo-5*-treated animals than the controls. Here, we report the highest concentration of glucose where only 6 ± 5% of the *elo-5*-treated animals were alive at day 4 compared with 82 ± 6% of the controls (Fig. 2*B*).

The fatty acid data generated from labeled N2 animals fed glucose showed a significant change in the provision of C17iso but not C15iso to the membrane. We therefore investigated whether depleting C17iso alone would result in altered survival in high-glucose conditions. To do so, we examined the role of ELO-6, which is only required for C17iso production (Fig. 2*A*). The *elo-6* RNAi-treated animals survived the glucose treatment similarly to wild-type animals with 81 ± 5% alive at day 4 (Fig. 2*B*) suggesting that the depletion of C17iso at least by 73% is not sufficient to cause death (Fig 2*C*). In support of this conclusion, the *elo-6* RNAi treatment reduces overall mmBCFA levels by 50%, while *elo-5* RNAi nearly eliminates the mmBCFAs completely (Fig 2*C*).

To confirm the role of mmBCFAs in directly impacting the response to glucose, we fed nematodes a diet containing 50% *Bacillus subtilis,* because this bacterial strain contains high levels of mmBCFAs. Consistent with our results indicating a role for mmBCFAs in responding to elevated dietary glucose, *B. subtilis* feeding restored the survival rates of animals on +gluc plates back to wild-type levels (Fig 2*D).* The levels of mmBCFAs in B. subtilis are 11.2 ± 0.3% C15iso and 5.2 ± 0.1% C17iso Fig 2*E*) compared with OP50 where mmBCFAs are completely absent (Fig 2*E*). Additionally, *B. subtilis* contains a high percentage of anteiso mmBCFA species that terminates in an isobutyl and not an isopropyl as in the iso species. Consistent with our results indicating a role for mmBCFAs in responding to elevated dietary glucose, *B. subtilis* feeding restored the survival rates of animals on +gluc plates back to wild-type levels (Fig. 2*D*). To ensure that this rescue of nematode viability with *B. subtilis* supplementation was not due to *elo-5* RNAi bacteria dilution, we fed wild-type nematodes a L4440/*elo-5* mixture to confirm sufficient RNAi knockdown with a diluted RNAi bacterial diet. Animals fed the L4440/*elo-5* mixture show high mortality under glucose stress, confirming our data presented previously (Fig. S1). Thus, *B. subtilis* can rescue phenotypes caused by the absence of ELO-5, and we conclude that the incorporation of new mmBCFAs from synthesis or from the diet is critical for glucose stress survival.

To further examine the relationship of mmBCFA in survival with glucose stress, we tested whether mmBCFA production was actively needed on +gluc plates or simply the presence of mmBCFAs synthesized prior to glucose exposure. We tested it by compromising mmBCFA synthesis only during adulthood and assessed the viability on +gluc plates. The *elo-5* knockdown was initiated concurrently with the glucose stress in day 1 adults. In the adult-only *elo-5* RNAi treatment, there is significantly reduced survival compared with controls with treated animals showing 50% of viability in day 9 as opposed to controls reaching 50% of viability in day 11 (Fig. 2*F*). The reduced survival on +gluc plates demonstrates that there is an active role for mmBCFA synthesis in responding to glucose that is independent of the abundance of mmBCFAs accumulated during development.

Finally, we confirmed that *elo-5 (gk208)* mutants also died prematurely on +gluc plates with 27% ± 10% alive at day 4 compared with 76 ± 5% alive in control animals (Fig. 2*G*). These experiments demonstrate for the first time a critical role for mmBCFAs in surviving glucose stress and elucidate a novel function for these unique fatty acids in adult animals.

### mmBCFAs act in the PAQR-2 network response to glucose stress

**Figure 3:**
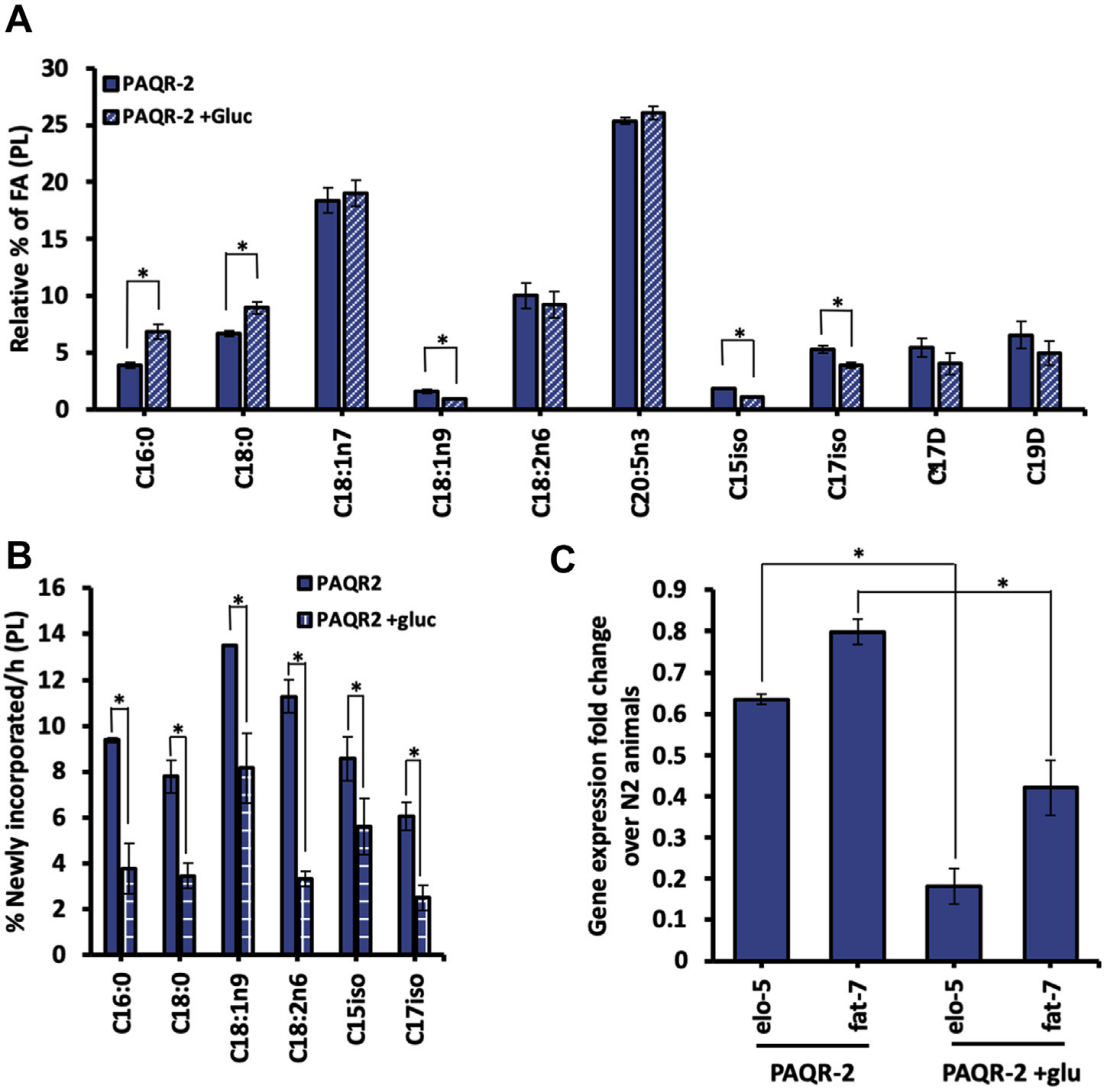
**PAQR-2 upregulates mmBCFA production under glucose stress.***A*, *paqr-2(tm3410)* mutants in the L4 stage (48 h old) were fed for OP50 bacteria seeded onto HG plates with (*blue stripe*) or without 15 mM of glucose (*blue*) for 12 h. Using GC/MS, the FA composition of PLs was assessed and significant changes were found in C16:0, C18:0, C18:1n9, C15iso, and C17iso as indicated by the ∗. *B*, a 6 h-period of isotope labeling shows a significant decrease in the amount of newly incorporated FAs (marked by ^13^C-isotopes) in all species quantified in *paqr-2* animals fed glucose (*blue checkered*). The relative % of FA and the % newly incorporated FA/h represent means ± SEM of at least three independent replicates. Statistical significance (*p* < 0.05) was calculated using unpaired *t* test and F test to compare variances. *C*, quantitative real-time PCR was implemented to quantify the transcript levels of *elo-5* and *fat-7* in *paqr-2* mutants compared with controls. Fold changes were quantified using the 2ˆ-ΔΔCt method, and significance was assessed by applying a One-Sample *t* test (hypothetical value = 1) on GraphPad Prism (v9.0.0). There is a significant reduction in expression of both *elo-5* and *fat-7* compared with N2 controls as denoted by ∗. mmBCFAs, monomethyl branched-chain fatty acids.

Previous studies characterize this seven-transmembrane protein PAQR-2 as an important membrane sensor that responds to decreased membrane fluidity resulting from excess glucose. Because of the high mortality of *elo-5* knockdown animals on +gluc plates (Fig. 2*B*), we hypothesized that the *elo-5* elongase could be activated by PAQR-2 as part of the metabolic response to glucose stress. To probe this relationship, we assessed the membrane composition of *paqr-2 (tm3410)* mutants immediately after glucose stress and analyzed their mmBCFAs abundance with GC/MS. We found significantly less mmBCFAs (C15iso and C17iso) in the membranes of *paqr-2* animals on +gluc plates compared with *paqr-2* animals on control plates (Fig. 3*A*). Specifically, C15iso was depleted from 1.7 ± 0.1% to 1.1 ± 0.1% and C17iso from 5.3 ± 0.3% to 3.0 ± 0.2% with glucose treatment, demonstrating that PAQR-2 may be required to maintain mmBCFA levels under glucose stress.

To further investigate the impact of PAQR-2 in the response to elevated glucose, we analyzed the membrane dynamics of *paqr-2* mutants with stable isotope labeling as previously described. In *paqr-2* mutant animals, there is a significant reduction in the labeling of mmBCFAs with C15iso levels decreasing from 8.6 ± 0.6% to 5.6 ± 0.7% and C17iso from 6.1 ± 0.3% to 2.5 ± 0.3%, indicating that PAQR-2 may be required for the upregulation of ELO-5. Furthermore, the analysis of the other fatty acid species in the membrane indicates a global reduction in the turnover of all species in *paqr-2* mutant animals on +gluc plates with the greatest decrease observed in linoleate (C18:2n6) (71% reduction) (Fig. 3*B*). It is important to note that the *paqr-2* mutants are unhealthy on glucose plates, so the reduction in turnover may be a consequence of their compromised health (13). However, PAQR-2 has been found to regulate *fat-7* expression, and reduction of *fat-7* by RNAi results in a global reduction of fatty acid flux, presumably to mitigate the impact of the compromised fatty acid pool on the membrane (15).

Since PAQR-2 is known to impact transcription of metabolic genes, we sought to examine the expression of *elo-5* in these mutants with and without glucose. First, we confirmed the requirement of PAQR-2 to induce *fat-7* expression with glucose and found that *fat-7* is expressed at 0.4 the levels of control animals (Fig. 3*C*). Interestingly, *fat-7* in PAQR-2 animals is at approximately 80% of wild-type expression in the absence of glucose. Next, we found that *elo-5* is only expressed at 60% of wild-type levels without glucose and 20% of wild-type levels with glucose (Fig. 3*C*). Overall, the qRT-PCR analysis demonstrates that *elo-5* is regulated by PAQR-2 and indicates that mmBCFA synthesis is a major factor in the PAQR-2 response.

**Figure 4:**
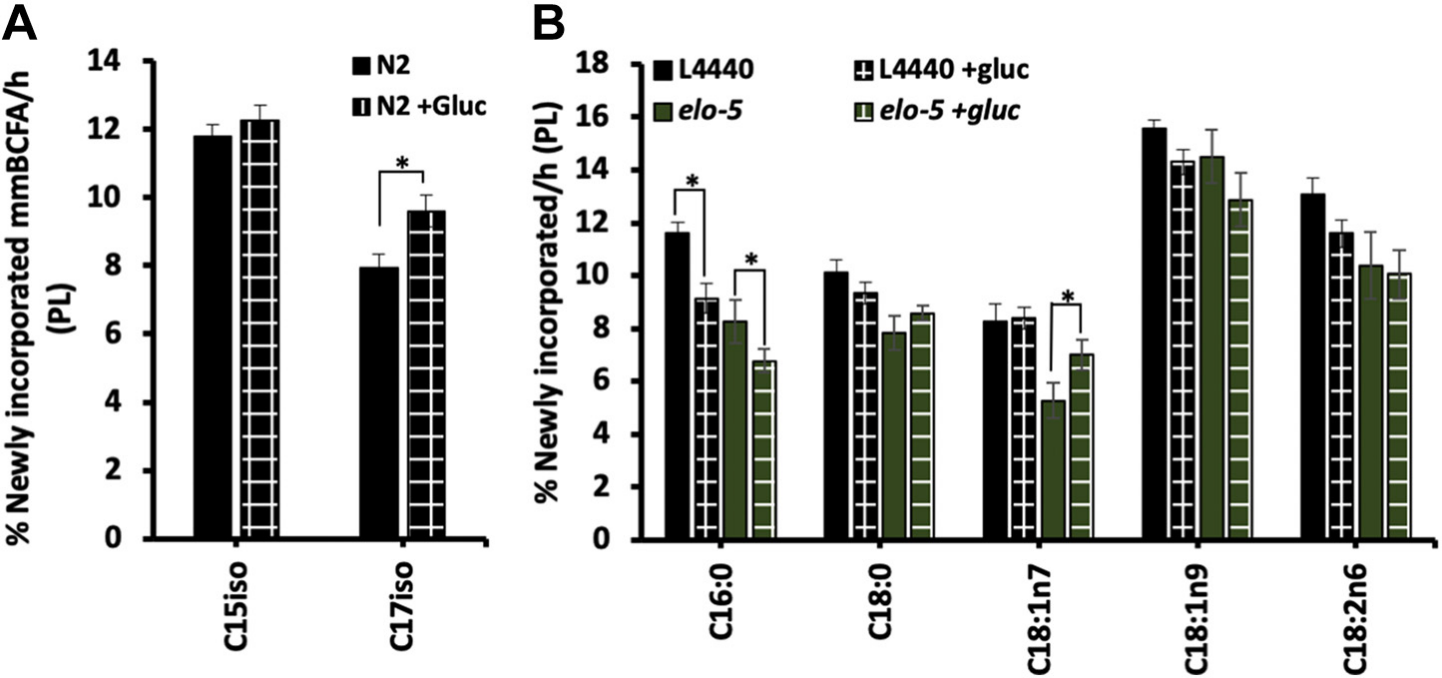
***elo-5* RNAi alters mmBCFAs dynamics in the membrane.***A*, the mmBCFA abundances are below the detection thresholds in *elo-5* RNAi but show the same increase in C17iso incorporation (*black checkered*) even with the altered food source (L4440 opposed to OP50). *B*, ^13^C-labeling of the same animals shown in graph C quantifies the amount of newly incorporated fatty acid and show significant decreases in C16:0 incorporation in both control (*black checkered*) and *elo-5* RNAi (*green checkered*) on +gluc. Additionally, there was a significant increase in newly incorporated C18:1n7 in *elo-5* +gluc. The relative % of FA and the % newly incorporated FA/h represent means ± SEM of at least three independent replicates. Statistical significance (*p* < 0.05) was calculated using an unpaired *t* test and F test to compare variances. mmBCFAs, monomethyl branched-chain fatty acids.

Finally, we used stable isotope labeling to confirm that the perturbation in mmBCFA production in the *elo-5* depleted animals does not impact the turnover of the other fatty acid species. The control for the RNAi studies is L4440 bacteria, which can carry the RNAi vector, and has a slightly different fatty acid composition. Therefore, we first confirmed the elevated mmBCFA turnover on +gluc with this diet and found that the wild-type animals fed L4440 showed high levels of turnover of C17iso on glucose compared with controls, confirming the mmBCFAs response seen previously in OP50-fed animals (Fig. 4*A*). In *elo-5* RNAi-treated animals, there is also a reduction in labeled C16:0 on +gluc plates, and although the initial abundance is lower, the decrease of 17.9% is consistent with control animals at 21.2% (Fig. 4*B*). Therefore, the nematode responds to the accumulation of SFA from glucose stress by downregulating the flux of C16:0 to the membrane but not through a pathway that requires ELO-5. Interestingly, unstressed *elo-5* animals start off with lower replenishment of C16:0 (Fig. 4*B*), indicating that the absence of *elo-5* enzymes impacts the flux of this SFA to the membrane even in control conditions.

In addition, the MUFA, vaccinate (C18:1n7), which is mostly provided by the diet, was significantly increased in *elo-5* knockdown nematodes (Fig S1). Because these animals lack mmBCFAs, the increase in C18:1n7 may alleviate the impact of this loss on the properties of the membrane. Taken together, our results indicate that a rewiring of FA metabolism pathways including mmBCFAs upregulation is essential to respond to glucose stress, and that C18:1n7 is a potential compensatory FA participating in the overall response.

### mmBCFAs act in parallel to the fluidity response in glucose stress

**Figure 5:**
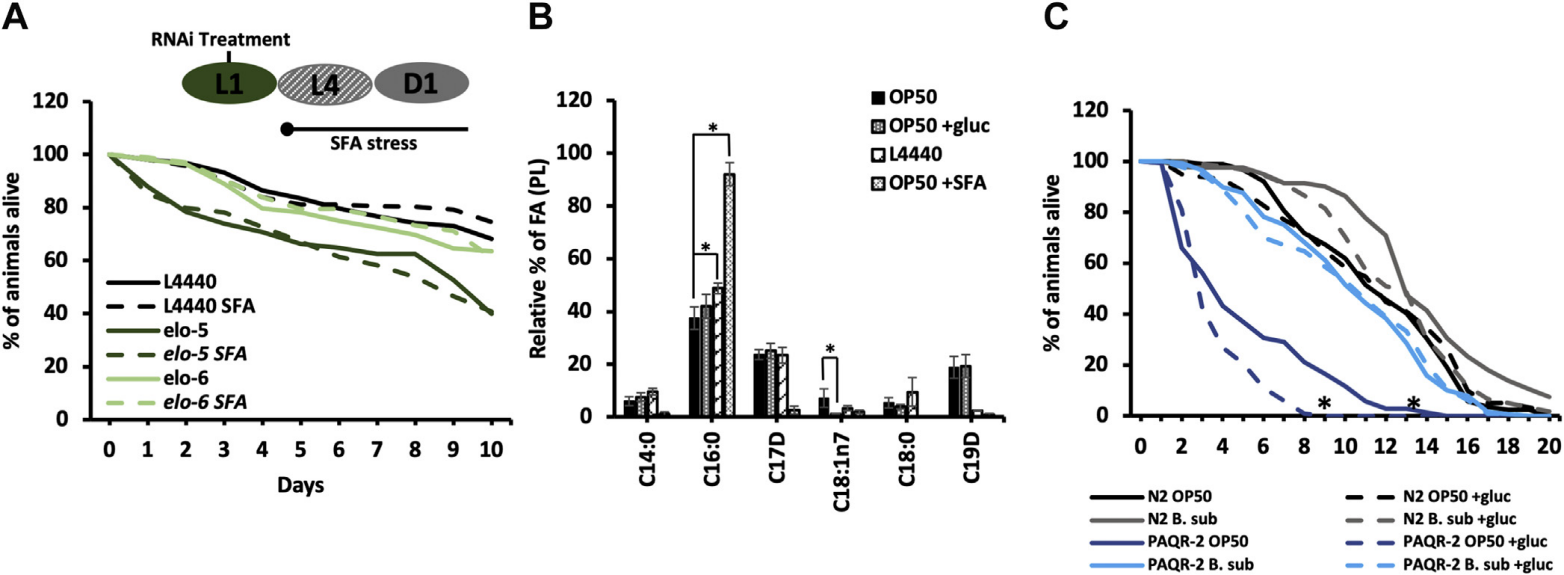
**mmBCFAs are not required to respond to elevated dietary saturated fatty acid.***A*, bacteria containing RNAi vectors (L4440, elo-5, and elo-6) were grown in LB + tetracycline + carbenicillin containing 2 mM of palmitic acid (C16:0), which is labeled as +SFA. RNAi knockdown of *elo-5* (*dark green*) and *elo-6* (*light green*) in N2 worms was initiated at L1 along with control empty vector RNAi. These animals were moved to +SFA plates (*dashed line*) at the end of the L4 stage, and the percent of animals alive was assessed daily. There was no significant difference in viability with RNAi treatment. *B*, GC/MS was used to assess the FA composition of the supplemented bacteria in comparison to the OP50 (*black*) and L4440 controls (*diagonal bricks*). There were slightly higher levels of SFA in the L4440 *versus* the OP50 control. Significantly higher levels of SFA were measured in the enriched OP50 +SFA (*white dotted*) as expected. Additionally, OP50 +glu (*gray dotted*) had significantly lower levels of C18:1n7. *C*, PAQR-2 mutants were fed *B. subtilis* (*blue*) in the presence of glucose (*dashed line*) and monitored daily. Compared with OP50-fed PAQR-2 (*dark blue*), there is a significant rescue in mean and maximal lifespan with *B. subtilis* feeding. There was no significant difference in the life span of N2 animals on *B. subtilis* with (*gray dashed*) or without (*gray solid*) glucose in comparison to N2 on OP50 ± glucose (*dark gray*). The replicates of the survival curves are presented as means of at least three independent replicates with n = 50/condition for each replicate. The statistical analysis of the survival curve was performed by the Log-rank (Mantel-Cox) test. *p* ≥ 0.0001. The lipid composition values represent means ± SEM of at least four replicates. Statistical significance (*p* < 0.05) was calculated using an unpaired *t* test and F test to compare variances. FAMEs, fatty acid methyl esters; mmBCFAs, monomethyl branched-chain fatty acids.

The major established role of PAQR-2 is to correct the reduced membrane fluidity that occurs with excess dietary glucose or excess dietary saturated fat. Because both C15iso and C17iso contain a branch within the hydrocarbon chain, these fatty acids disrupt lipid packing and promote fluidity within a membrane. In order to determine if mmBCFAs play a general role in restoring membrane fluidity, nematodes were fed excess SFA (2 mM C16:0) and examined for altered development and survival. First, we investigated whether ELO-5 is required for growth and development on plates with excess SFA (2 mM C16:0), which we refer to as +SFA plates. There was no impact on larval development in *elo-5* RNAi fed animals on +SFA in contrast to the arrest seen in *paqr-2 (tm3410)* animals (Fig. S1). Next, the mortality rates of *elo-5* and *elo-6* RNAi-treated animals were compared with controls. The incorporation of exogenous SFA did not impact viability of *elo-5* or *elo-6* RNAi-treated animals (Fig. 5*A*). The lack of impact with SFA supplementation demonstrates that the role of mmBCFAs is not in a general response to membrane saturation but specific to certain conditions such as high glucose.

To confirm SFA incorporation, we quantified the amount of C16:0 in the phospholipid population of the supplemented animals and found a significant increase in C16:0 abundance from 3.8% to 5.5% in wild type and from 3.6% to 6.2% in *elo-5* RNAi knockdown (Fig. S1). We also confirmed a significant increase of C16:0 in the bacteria on the +SFA plates and found that this diet contains 92% SFA (Fig. 5*B*). In order to determine how the C16:0 was incorporated into the nematode, the fatty acid population of neutral lipids was also measured, and there was an increase from 7.7 ± 0.8% to 15.6 ± 9.3% in wild-type animals (Fig. S1). Not only does this data confirm that significant C16:0 is available for the nematodes, but it also demonstrates active mechanisms to protect the membrane from excess saturated fat. We can also conclude that the role of mmBCFAs is not in reestablishing membrane fluidity.

Because our earlier experiments suggest that PAQR-2 may coordinate the response in mmBCFAs, we tested whether the mmBCFA-enriched *B. subtilis* could improve survival of *paqr-2* animals on +gluc plates. We found that the *B. subtilis* diet dramatically extended the life span of *paqr-2* animals on +gluc, further suggesting that the mmBCFAs are a critical component to the metabolic response to elevated glucose (Fig. 5*C*). Moreover, we observed that *paqr-2* animals had a compromised lifespan even in the absence of glucose with a mean life span of 3.5 ± 1 days compared with N2 on OP50 with a lifespan of 11.5 ± 2 days. A shortened life span has been reported for *paqr-2* animals previously; however, the difference was not as dramatic as shown here perhaps due to changes in the dietary fatty acid composition. Despite the short life span, we find that the addition of +gluc plates further compromises the life span of *paqr-2* animals to 8 days. Taken together, these experiments strongly implicate mmBCFAs as part of the PAQR-2-driven response to elevated dietary glucose.

## Discussion

**Figure 6:**
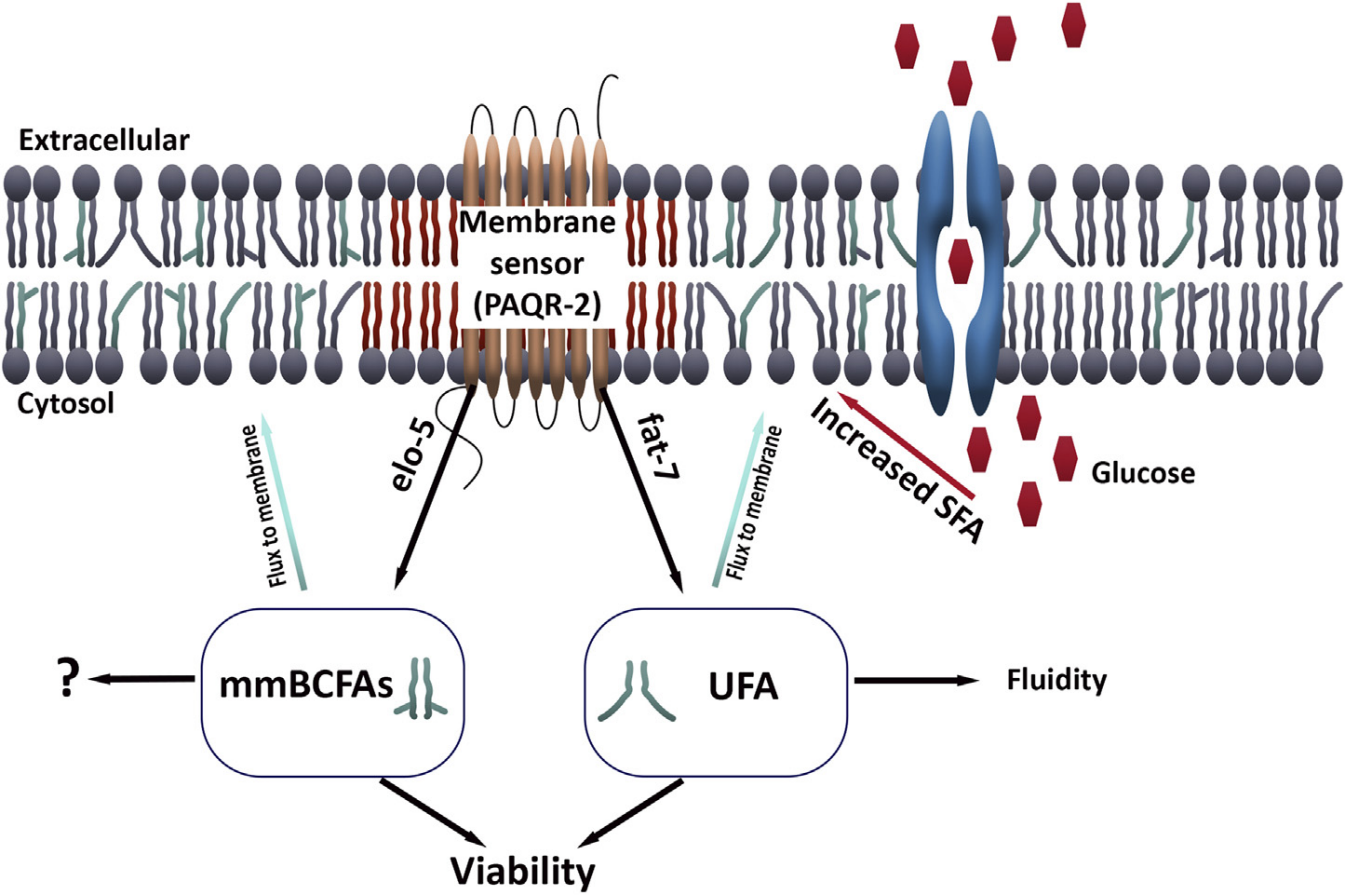
**mmBCFAs play a role in the membrane’s response to glucose stress.** Our model for the role of mmBCFA in elevated glucose conditions is shown here. First, elevated dietary glucose decreases the fluidity of the membrane in nematodes and drives a response coordinated by PAQR-2. Next, PAQR-2 impacts the transcription of genes including *elo-5* and *fat-7* to restore membrane integrity and to promote survival under glucose stress. Unlike unsaturated fatty acids, the role of mmBCFAs is not to restore overall fluidity but instead this fatty acid may be involved in signaling. mmBCFAs, monomethyl branched-chain fatty acids.

We aimed to identify mechanisms that provide an effective response to elevated dietary glucose conditions that perturbs the optimal membrane composition when unaddressed. Although it is known that the PAQR-2 membrane sensor responds to glucose stress through the FAT-7 desaturase, we utilized a stable isotope labeling approach to identify a new component of the PAQR-2 response (Fig. 6). Specifically, our labeling studies identified that under glucose stress, the membrane receives significantly lower levels of palmitic acid and higher levels of C17iso. The upregulation of mmBCFA production through ELO-5 is critical for survival on glucose as *elo-5* RNAi results in severely compromised survival on +gluc plates. Our results confirm that we have identified mmBCFAs in a novel and essential response to elevated glucose.

Previous FRAP studies have shown that elevated glucose decreases membrane fluidity and have found an increased incorporation of saturated fat into the membrane lipids (31). It has been suggested that the elevated saturated fat is produced by the bacteria fed surplus glucose through *de novo* fatty acid synthesis. However, we quantified the saturated fat abundance from the glucose-fed bacteria (OP50+gluc) and found it has a slight but insignificant increase in C16:0, suggesting that the increase in SFA may be occurring in the nematode. In addition, glucose-stressed animals have a distinct response and life span compared with SFA stress, because we found ELO-5 is required for survival on glucose but not SFA. It is possible that the decreased fluidity in glucose stress could be due to an imbalance between the SFA and UFA, because the OP50 + gluc diet has significantly lower levels of C18:1n7. Since this fatty acid is mostly obtained from diet (14), the nematode would incorporate a disproportionately lower amount of MUFA, which would impact the membrane fluidity. However, the data thus far favors the hypothesis that the surplus glucose is influencing the animals independent of saturated fatty acid accumulation.

In nematodes, mmBCFAs have been shown to be involved in postembryonic growth (18), sensory neuron maturation, and foraging (20). However, mmBCFAs can impact the membrane directly, as mmBCFAs influence overall membrane fluidity since the branch in their structure prevents tight lipid packing. Because excess dietary SFA decreases membrane fluidity but the loss of *elo-5* does not exacerbate this phenotype, we hypothesize that the role of mmBCFAs is not in regulating membrane fluidity. Instead, a study conducted by Zhu *et al* found that the mmBCFA-derived sphingolipid d17iso-Glucosylceramide was linked to the target of rapamycin-1 (TORC1) (21). The GlcCer/Torc1 pathway was proposed to coordinate metabolic status in the intestine with growth and postembryonic development (32, 33). Therefore, the elevated levels of mmBCFAs observed in high-glucose diet may serve to increase the induction of TORC1 signaling to then increase the uptake and metabolism of glucose, which will be a direction of future study.

In wild-type animals, there is a significant upregulation of C17iso but not C15iso as revealed by stable isotope tracing. Interestingly, on +gluc plates, there are no glucose sensitivity in the *elo-6* RNA, which depletes C17iso but not C15iso levels. The lack of *elo-6* phenotypes may indicate a compensatory response by C15iso when C17iso synthesis is not available as there are still significant levels of C15iso in those treated animals. We propose that the role of mmBCFAs is not specific to one type of fatty acid but to the overall abundance of the mmBCFA population as there are substantially less mmBCFA in *elo-5* RNAi-treated animals than in *elo-7* RNAi-treated animals. This hypothesis is supported by the fact *elo-5* RNAi can be rescued by *B. subtilis*, which predominantly contains anteiso mmBCFAs suggesting that the specific identity of the mmBCFA may not be critical.

It is clear that PAQR-2 plays an important role regulating downstream desaturases to maintain SFA:UFA balance and consequently membrane fluidity; however, analyzing the membrane composition of PAQR-2 mutants revealed a significant decrease in the abundance of mmBCFAs. This suggests that PAQR-2 may also regulate ELO-5 to produce mmBCFAs in response to glucose stress. We were unable to determine if mmBCFAs were specifically compromised in PAQR-2 animals, because our isotope labeling analysis showed a global downregulation in the lipid renewal of all fatty acid species measured. The global decrease in lipid rejuvenation is consistent with the role of PAQR-2 in regulating FAT-7, which we have previously established is required for normal rates of lipid replacement. However, it is also possible that PAQR-2 animals have reduced food intake due to compromised health. Although it was shown previously that PAQR-2 mutants have a short life span (34), our data appear to show a more drastic life span impact compared with published studies. We hypothesize that the high mortality might be related to differences in the food sources provided to the worms, since PAQR-2 mutants seem to be strongly affected by the dietary content. In addition to the quantification of lipid metabolism in *paqr-2* animals, we found that *B. subtilis,* a diet rich in mmBCFAs, can rescue not only *elo-5* animals but also *paqr-2* mutants even without glucose exposure. This rescue supports the conclusion that ELO-5 is downstream of PAQR-2.

The identification of ELO-5 as essential for surviving excess dietary glucose required the use of stable isotopes, because GC-MS measurements did not reveal this requirement. Although this finding highlights the unique capabilities of the stable isotope feeding approaches, it is important to note that this technique has some limitations. The stable isotope technique cannot be used to detect PUFA species with more than two double bonds, because it relies on the detection of parent ions, and PUFAs fragment extensively under GC/MS conditions, yielding unreliable results. Also, current stable isotope labeling studies require quantification of the membrane in whole animals to generate sufficient biomass, and it is possible that the response observed could be tissue or organelle specific. Indeed, it has been shown that mmBCFAs are regulated distinctly in different tissues. In mice, the mmBCFAs were ten times more abundant in adipose tissue compared with liver cells (22). The exploration of the role of mmBCFA in individual tissues will be the focus of future work. Additionally, it will be critical to establish the relationship between PAQR-2 and mmBCFAs in more detail and to identify how mmBCFAs impact survival with glucose. In summary, we have identified a novel role for mmBCFAs under elevated dietary glucose using stable isotopes and mass spectrometry.

## Experimental procedures

### *C. elegans* and bacteria growth and maintenance

Unless noted, the experiments were conducted using wild-type N2 nematodes obtained from the *C. elegans* Genetics Center (CGC). *paqr-2 (tm3410)* and *elo-5* (gk208) were obtained from the *C. elegans* Genetics Center (CGC). In order to synchronize the nematodes, gravid adults were exposed to dilute bleach, and the washed eggs were left rotating overnight at 20 °C in M9 solution. Unless specified, OP50 bacteria on HG plates were used to feed the nematodes. For knockdown experiments, RNAi strains L4440 (empty vector), *elo-5,* and *elo-6* from the Ahringer library were grown on NGM + Carbenicillin + IPTG plates (NGM+CI) (35). *B. subtilis* (*sp168*) was provided by the Walhout lab at UMMAS and was cultivated in the same media and following the same protocols used with OP50.

### Altered dietary conditions

The +gluc plates were made to a final concentration of 15 mM glucose or 45 mM glucose by adding a filtered glucose solution to cooled autoclaved HG media. The +gluc plates were seeded with regular OP50 bacteria at least 4 days before plating the worms. Synchronized L4 stage worms grown on HG plates were transferred to +gluc plates for 12 h. After the glucose exposure, animals were labeled using ^13^C stable isotopes as described in the following section. For C16:0 stress, L4 animals were transferred to NGM-CI plate (without peptone) inoculated with control or RNAi bacteria. To supplement the SFA in the diet, 2 mM of C16:0 was added to the media before the bacteria were inoculated (31). After 18 to 20 h growth, the bacteria was washed three times with fresh liquid media and resuspended in LB or LB+carb+tet liquid media and spread on the appropriate plate.

RNAi bacteria along with the control RNAi were plated at a density of 0.15 g per 10 cm NGM-CI plate. Approximately 8000 to 10,000 synchronized L1 N2 animals were grown at a density of 2500 worms per RNAi treatment plate for 48 h. At L4, the nematodes were transferred to +gluc plates for 12 h.

### Viability curves and life spans

To quantify survival on +gluc plates, RNAi bacteria (L4440, *elo-5, elo-6*) were seeded onto 3 cm NGM +CI plates, and L1 worms were grown for 48 h. Approximately 50 L4 stage nematodes were then transferred to fresh NGM +CI +gluc plates each day, and the number of dead animals was determined by gently prodding with a pick. *B. subtilis* supplementation started at L1 stage in a mixture prepared with RNAi bacteria (50%:50%) and then continued as described with the RNAi bacteria.

### Stable isotope labeling strategy

Animals were labeled with isotopes following the protocols established in (14). Briefly, Isogro media (^13^C) and LB media (^12^C) were inoculated with OP50 colonies to allow the growth of bacteria for 16 h at 37 °C. Next, the bacteria were harvested and resuspended in M9 in the concentration of 015 g/ml. A mixture containing enriched bacteria ^13^C:^12^ C (50%:50%) was transferred to agarose plates and allowed to dry. Nematodes from +gluc plates were harvested, washed three times using M9, and plated onto stable isotope labeling plates containing 800uL of bacteria mixture for 6 h. The exact ratio of ^13^C:^12^ C was quantified by GC-MS. Labeled worms were harvested, washed, and stored at −80 °C until lipid extraction and analysis by GC/MS.

### qRT-PCR testing

The RNAs were extracted using Direct-zol RNA Miniprep kit (Zymo Research Corporation, Cat# R2050) following the manufacturer's instructions. In total, 500 ng of RNAs was reverse-transcribed using M-MuLV reverse transcriptase (NEB, Cat# M0253S) following the manufacturer's instructions. The resulting cDNAs were diluted ten times, and 2 μl was used for 20 μl qPCR reactions, performed using Fast SYBR Green Master Mix (Thermo Fisher Scientific, Cat# 4,385,612) following the manufacturer's instructions, on a QuantStudio 3 Real-Time PCR System (Thermo Fisher Scientific, Cat# A28567). Fold changes were quantified using the 2ˆ-ΔΔCt method, and significance was assessed by applying a One-Sample *t* test (hypothetical value = 1) on GraphPad Prism (v9.0.0).

Primer sequences:

**Table:** 

Fat-7-298R:	Accagagacgatgggctccagc
fat-7-195F:	Cgtcgccgcagccattggactt
fat-6-490R:	ctccctctccgacggcagcaat
fat-6-407F:	tggtgcatcaacagcgctgctca
fat-5-795R:	acctccttctccgactgccgca
fat-5-667F:	acggccgccctcttccgttact
elo-6-156R:	tgtcatgacagccttgagccca
elo-5-159R:	actgagatcgaaggcttttcggt
elo-6-48F:	tgaggtgctgacaactgctcca
elo-5-64F:	tgccaaagaagttgctcgaggcc

### Lipid extraction and GC analysis

Total lipid was extracted, and PL and NL populations were separated using solid-phase chromatography for GC-MS (14, 15). Briefly, internal lipid standards, 1,2-diundecanoyl-sn-glycero-3-phosphocholine (Avanti Polar Lipids) and tritridecanoin (Nu-Chek Prep), were added to each sample, and total worm fat was extracted with chloroform:methanol (2:1) for 1.5 h at room temperature. Dried total lipids in 1 ml of chloroform were loaded onto HyperSep Silica SPE columns (100 mg capacity, Thermo Scientific), and NLs and PLs were collected. Purified PLs and NLs were dried and resuspended in 1 ml of 2.5% H_2_SO_4_ in methanol, then incubated for 1 h at 80 °C to create fatty acid methyl esters (FAMEs) to run on GC-MS (Thermo trace 1310, ISQ LT single quadruple).

We calculate the Relative % of FAs in each sample by using the integrated area under the peaks of each fatty acid species seen in the gas chromatograph and as defined here: Relative FA Abundance = FA area/Total FA area ∗100 as in (14). Labeling with stable isotopes (^13^C) allows for the analysis of the percentage of newly incorporated or Mol Percent Excess (MPE) of most major FA species simultaneously. Briefly, to calculate the MPE, the isotopomers were normalized and corrected to the incorporation of natural isotopes. % Newly Incorporated Fatty Acids consider all newly modified fat independent of its source (*de novo* synthesized, elongated or directly absorbed) as described (Dancy *et al*). Error bars of the lipid composition and ^13^C labeling show the standard error of the mean, and *t-tests* were used to identify significant differences between the fatty acids.

## Data availability

All data generated in this study is available in the article.

## Supporting information

This article contains supporting information.

## Conflict of interest

The authors declare that they have no conflicts of interest with the contents of this article.

## Figures and Tables

**Figure S1:**